# Artificial Intelligence Algorithms and Their Current Role in the Identification and Comparison of Gleason Patterns in Prostate Cancer Histopathology: A Comprehensive Review

**DOI:** 10.3390/diagnostics14192127

**Published:** 2024-09-25

**Authors:** Usman Khalid, Jasmin Gurung, Mladen Doykov, Gancho Kostov, Bozhidar Hristov, Petar Uchikov, Maria Kraeva, Krasimir Kraev, Daniel Doykov, Katya Doykova, Siyana Valova, Lyubomir Chervenkov, Eduard Tilkiyan, Krasimira Eneva

**Affiliations:** 1 Medical Faculty, Medical University of Plovdiv, 4002 Plovdiv, Bulgaria; usmankhalid957@gmail.com (U.K.); jasmingurung12@gmail.com (J.G.); 2 Department of Urology and General Medicine, Medical Faculty, Medical University of Plovdiv, 4002 Plovdiv, Bulgaria; mladen.doykov@mu-plovdiv.bg; 3 Department of Special Surgery, Faculty of Medicine, Medical University of Plovdiv, 4002 Plovdiv, Bulgaria; gancho.kostov@mu-plovdiv.bg (G.K.); puchikov@yahoo.com (P.U.); 4 Second Department of Internal Diseases, Section “Gastroenterology”, Medical Faculty, Medical University of Plovdiv, 4002 Plovdiv, Bulgaria; hristov.bozhidar@abv.bg (B.H.); daniel_doykov@abv.bg (D.D.); 5 Department of Otorhinolaryngology, Medical Faculty, Medical University of Plovdiv, 4002 Plovdiv, Bulgaria; kraevamaria93@gmail.com; 6 Department of Propedeutics of Internal Diseases, Medical Faculty, Medical University of Plovdiv, 4002 Plovdiv, Bulgaria; 7 Department of Diagnostic Imaging, Medical Faculty, Medical University of Plovdiv, 4002 Plovdiv, Bulgaria; katia_doykova@abv.bg (K.D.); lyubo.ch@gmail.com (L.C.); 8 Second Department of Internal Diseases, Section “Nephrology”, Medical Faculty, Medical University of Plovdiv, 4002 Plovdiv, Bulgaria; siyanavalova@abv.bg (S.V.); eet64@yahoo.com (E.T.); 9 Department of Infectious diseases, Parasitology and Tropical medicine, Medical University of Plovdiv, 4002 Plovdiv, Bulgaria; kr_eneva@abv.bg

**Keywords:** artificial intelligence, Gleason pattern, prostate cancer, histopathology

## Abstract

The development of the Gleason grading system has proven to be an irreplaceable tool in prostate cancer diagnostics within urology. Despite the advancements and developments in diagnostics, there remains a discrepancy in the grading process among even the most experienced pathologists. AI algorithms have demonstrated potential in detecting cancer and assigning Gleason grades, offering a solution to the issue of significant variability among pathologists’ evaluations. Our paper explores the evolving role of AI in prostate cancer histopathology, with a key focus on outcomes and the reliability of various AI algorithms for Gleason pattern assessment. We conducted a non-systematic review of the published literature to examine the role of artificial intelligence in Gleason pattern diagnostics. The PubMed and Google Scholar databases were searched to gather pertinent information about recent advancements in artificial intelligence and their impact on Gleason patterns. We found that AI algorithms are increasingly being used to identify Gleason patterns in prostate cancer, with recent studies showing promising advancements that surpass traditional diagnostic methods. These findings highlight AI’s potential to be integrated into clinical practice, enhancing pathologists’ workflows and improving patient outcomes. The inter-observer variability in Gleason grading has seen an improvement in efficiency with the implementation of AI. Pathologists using AI have reported successful outcomes, demonstrating its effectiveness as a supplementary tool. While some refinements are still needed before AI can be fully implemented in clinical practice, its positive impact is anticipated soon.

## 1. Introduction

The development of the Gleason grade has proven to be an irreplaceable tool in prostate cancer diagnostics within urology. Developed in 1966 by Donald Gleason, it has gained almost universal acceptance by facilitating risk communication and enhancing treatment decision-making [1,2]. The Gleason grade assesses the architecture or arrangement of the malignant cells and incorporates factors, such as the degree of differentiation [3]. The Gleason score is calculated by summing two numbers, each highlighting different Gleason grades. The first number depicts the grade of the primary cancer cell pattern, and the second number is the grade of the next most common pattern. When only one Gleason grade is present, it is doubled to form the score. This score is typically written in a format like 3 + 4 = 7. In this example, the first 3 is the most common grade while the 4 shows the second most common grade, giving a total Gleason score of 7.

However, the formula would be 4 + 3 = 7 if the predominant pattern was grade 4. Even though the total score remains 7, this configuration indicates a more aggressive cancer. If there is a third, higher-grade, pattern, it replaces the secondary grade in the score calculation. A Gleason score of less than 6 usually indicates a state of indolence within the cancer, which constitutes a lack of clinical significance, but scores including 8 or above are frequently related to poor tumor differentiation and an unfavorable prognosis [4].

In 2014, a revision of the Gleason scoring method was performed at the International Society of Urological Pathology consensus meeting. The revisions included the percentage of Gleason pattern 4 and the introduction of five new grades of prostate cancer, determined by the Gleason score. Under the new grading system, Grade I consisted of Gleason scores of 6 and below, with Grade II being defined by a Gleason score of 3 + 4 = 7. Grade III included a Gleason score of 4 + 3 = 7, Grade IV with a Gleason score of 4 + 4 = 8, and finally Grade V, which is composed of all Gleason scores between 9 and 10 [5].

Artificial intelligence (AI) focuses on creating autonomous systems capable of performing tasks traditionally carried out by humans, employing advanced non-linear mathematical simulations and simple components that mimic human neurons. This field starts by exploring how the human mind perceives, understands, and executes cognitive functions, such as intelligence, creativity, language comprehension, memory, pattern recognition, vision, reasoning, and connecting information. AI aims to replicate these abilities to handle a range of activities, from straightforward tasks, like object recognition, to more complex functions, such as forecasting.

AI strategies involve learning from existing data without inherent biases, relying on statistical models, and predicting future outcomes. This approach enhances decision-making by making it more informed and efficient.

The aim of AI is to develop machines that can sense their surroundings and perform actions to optimize their success. Achieving this involves various subfields, including machine learning (ML), artificial neural networks (ANNs), deep learning (DL), natural language processing (NLP), computer vision, predictive analytics, evolutionary and genetic algorithms, expert systems, and speech processing [6].

In the healthcare sector, artificial intelligence (AI) encompasses a wide range of applications, systems, algorithms, and devices designed to assist healthcare providers by leveraging computer systems and big data. Medical data are used to support doctors and patients in making informed decisions and selecting the most appropriate treatments. AI plays a crucial role in developing innovative methods for analyzing complex, data-intensive tasks, utilizing various AI disciplines. In addition to enhancing patient care, AI also boosts efficiency in research and development (R&D) and helps identify disease patterns and correlations earlier than traditional methods [7].

Recently, there has been a significant surge in investment and interest in AI applications within medicine, driven by growing evidence that AI can improve healthcare delivery.

Despite the advancements and developments in diagnostics, there is still a discrepancy in the grading process among the most experienced [8]. The fruitful rise of AI has paved the way for enhanced medical diagnostics. Recent research has significantly advanced the diagnosis of tumors across various organs, with a notable focus on prostate cancer. The integration of deep learning (DL) with histopathology and radiology, particularly magnetic resonance imaging (MRI), has become crucial in the grading of prostate cancerous tissues [9]. This combination leverages the strengths of both imaging modalities to enhance diagnostic accuracy and improve the assessment of cancer severity. The emergence of digital pathology has enabled the creation of AI systems designed to assist pathologists in analyzing prostate biopsies. These AI tools have demonstrated potential in detecting cancer and assigning Gleason grades, offering a solution to the variable inconsistencies which lie within the pathologists’ assessments [10].

## 2. Aim

This paper aims to explore the present role of artificial intelligence for targeted prostate cancer histopathology by assessing and comparing the outcomes and reliability of multiple AI algorithms geared toward Gleason pattern detection.

## 3. Method

We conducted a non-systematic review of the existing literature on the application of artificial intelligence in diagnosing Gleason patterns. We explored the PubMed and Google Scholar databases to gather insights on the latest advancements in AI and their effects on Gleason pattern analysis. Various keyword combinations were employed to pinpoint pertinent studies published up to 3 July 2024. Some keywords include artificial intelligence, Gleason pattern, prostate cancer, and histopathology. Only those articles matching our search criteria were considered relevant. Additionally, we excluded any articles not published in English from our review.

The literature review was conducted according to established inclusion criteria. Initially, titles and abstracts were evaluated, followed by a thorough review of the full text of selected articles based on these criteria. Additionally, the reference lists of the chosen articles were manually reviewed to identify any relevant supplementary studies. Any disagreements about article eligibility were resolved through discussion among the authors, leading to a consensus decision.

Inclusion Criteria:

1. Articles related to artificial intelligence in urology;
2. Review papers and abstracts;
3. Original articles of full-text length, covering the diagnoses, treatment plans, and results of urologic conditions.

### 3.1. Machine Learning Approaches for Automated Gleason Pattern Classification

Automation is an area of machine learning that allows the identification and categorization of sections into their respective divisions. With highly sensitive and specific networks being incorporated into Gleason-grade identification, the role of automated learning has been given a spotlight. Ao et al. demonstrated that stimulated Raman scattering (SRS) microscopy built on a deep learning model can automate diagnoses and sort needle biopsy prostatic tissue according to Gleason scores. A diagnostic (convolutional neural network) CNN trained on images from 61 patients achieved 85.7% accuracy in classifying Gleason patterns of prostate cancer. Validation with an additional 22 independent cases yielded 84.4% accuracy. Using the deep learning-stimulated Raman scattering system, Gleason scores for core needle biopsies from 21 cases showed 71% diagnostic consistency among 3 pathologists. With timely histopathology-compatible evaluations for FT therapeutics, the study underscores the ability of deep-learning assisted SRS platforms to simplify prostate cancer diagnosis [11].

A separate study compared an automated tumor assessment of prostate cancer histology (ATARI) to a residual network with 101 layers of automated Gleason grading [12]. They hypothesized that a machine learning model utilizing second-order features from digitized histology images could distinguish prostate cancer from normal tissue. Additionally, they posited that a deep learning model would show varying classification accuracy in both cancer detection and Gleason pattern differentiation. The results demonstrated that the ensemble and ResNet models produced overall accuracies of 89% and 88%, respectively, in identifying cancer versus non-cancer. Notably, only the ResNet model could distinguish Gleason patterns in Cohort B. These findings indicate that while quantitative pathomic features can identify cancer regions, deep learning models excel at distinguishing specific Gleason patterns [13].

Ramamurthy et al. proposed a deep learning network based on EfficientNet architecture that balances the underlying network dimensions and applies a compound scaling method. The models were developed using H&E-stained tissue microarrays (TMAs) of prostate cancer from the Harvard Dataverse dataset. This network surpassed existing methods, achieving a kappa score of 0.5775 [14]. Bhattacharya utilized CorrSigNIA, which integrates aligned MRI and whole-mount histopathology images from radical prostatectomy patients to generate precise ground truth labels and identify correlated features between radiology and pathology images.

This study targeted pathological characteristics identified in resected tissue that do not differentiate between aggressive cancers (Gleason pattern ≥ 4) and indolent cancers (Gleason pattern = 3) when they are present in mixed lesions. With an accuracy of over 80%, CorrSigNIA performed exceptionally when differentiating between cancer and non-cancer cases, achieving an AUC of 0.81 ± 0.31 for cancer identification within biopsy cohorts and radical prostatectomy. Further results yielded an AUC of 0.82 ± 0.31 and 0.86 ± 0.26 when identifying clinically significant cancers in these cohorts, respectively

CorrSigNIA consistently outperformed other methods across various metrics and datasets.

In clinical practice, it can enhance prostate cancer detection and distinguish between indolent and aggressive cancer components, thus aiding in targeted biopsies, minimizing unnecessary procedures, and optimizing treatment planning [15]. Using digitized prostate biopsy specimens, a computer aided diagnostic (CAD) system was developed for the classification of automated grade groups, which would also classify the Gleason pattern and the Gleason score. 

Among the three CNN models, CNNL achieved the highest patch classification accuracy at 0.76. The macro-averaged and weighted-average metrics spanned between 0.70 and 0.77. For the Gleason grade (GG) classification, the CAD system demonstrated an accuracy of around 80% and recall for the Gleason grade (GG) classification, with F1 scores ranging from 60% to 80%. To reinforce this, the precision and negative predictive value (NPV) were approximated at almost 94%. These results were assessed against standard models like ResNet50 and VGG-16 to emphasize the superior performance of the CNN in classifying patches. The results from the proposed pipeline demonstrate its capability to classify all five Gleason grades of prostate biopsy samples, outperforming standard CNNs. The agreement between the CAD system and pathologists is on par with the inter-rater reliability observed among pathologists [9].

### 3.2. Advanced Neural Networks for Gleason Pattern Recognition

Although the Gleason grading system is essential for managing prostate cancer, it presents several challenges. The process is labor-intensive, time-consuming, and often subject to significant variability among observers. Within this, there are often differing assigned grades between core biopsies and prostatectomy samples, as core biopsies may not fully capture the extent of the cancer [16]. These problems highlight the need for more consistent and efficient methods of grading prostate cancer. With its capability to transform visually intensive fields, AI has the potential to enhance both the speed and precision of prostate cancer grading.

Among the various neural networks, South Korean-based DeepDx is a prostate AI algorithm that employs deep neural networks to identify and systematically grade prostate cancerous tissue. With the algorithm trained and validated on 1133 prostate core needle biopsies, it could identify and differentiate between non-cancerous and cancerous tissue, highlighted by a Cohen’s kappa score of 0.91 in agreement with the reference standard.

For distinguishing malignant from benign cases, the algorithm performed with accuracy, sensitivity, specificity, and a positive predictive value (PPV) of 96%, 99.70%, 88%, and 95%, respectively. The consistency of the results was maintained when discriminating between Grade Group (GG) ≥ 2 versus GG 1 and benign cases with an accuracy of 92%, a sensitivity of 98%, a specificity of 85%, a PPV of 88%, and an NPV of 97%. When identifying GG ≥ 3 from GG 1–2 and benign cases, DeepDx prevailed, with results showing 92% accuracy, 95% sensitivity, 90% specificity, 87% PPV, and 96% NPV. The DeepDx Prostate AI algorithm has demonstrated excellent potential to identify and grade prostate cancer in digital histopathology images of whole-mount radical prostatectomy (RP) specimens. It has demonstrated high levels of agreement with expert GU pathologists and performs well in critical clinical tasks [17].

An alternative study found an improvement in the diagnostic accuracy for pathologists who incorporated DeepDx, leading to an enhanced concordance relative to pathologists who didn’t use the algorithm. This was seen more specifically in Gleason patterns 4 and 5 where the kappa value was elevated from 0.741 to 0.925 and the quadratic weighted kappa also saw a rise from 0.621 to 0.876 with the incorporation of network. With a diagnostic time reduced by 33.9%, the combination of these results highlights the precision and efficiency of DeepDx in high-grade prostate cancer detection [18].

With 1133 cases of prostate core needle biopsy, Ryu et al. trained the same neural network. They found a high level of diagnostic agreement between the system’s grade group classification and the reference standard, as shown by the quadratic-weighted Cohen’s kappa coefficient of 0.907. Additionally, the system’s tumor length measurements demonstrated a stronger correlation (r = 0.970) with the reference standard compared to the original hospital diagnoses (r = 0.900). When evaluating prostate biopsies, the system has the potential to assist pathologists in reducing the risk of overdiagnoses or underdiagnoses by offering second opinions on the Gleason score with pathologist-level accuracy. Furthermore, it aims to support research on prostate cancer treatment and prognosis by providing reproducible diagnoses based on consistent standards [19].

U-Net is another deep neural network utilized for the effective and accurate diagnosis of prostate cancer, most specifically with prostate adenocarcinomas [20]. When evaluated on whole image testing, the AI system and pathologists produced identical diagnoses for 21 out of 22 sections. When given the task of distinguishing cancerous from non-cancerous areas, the AI system achieved an AUV of 96.8%, with the pixel accuracy across three conditions maintaining an accuracy of 95.43% in clinically orientated analysis, 96.93% in binary analysis, and 93.88% in different ISUP Gleason grades.

Additionally, the frequency-weighted Intersection over Union (IoU) values were 94.32% for binary analysis, 92.13% for clinically oriented analysis, and 90.21% for ISUP Gleason grade analysis, reinforcing the AI system’s robust capabilities in assisting pathologists make to a final decision [21].

To tackle inter-observer variability when evaluating the Gleason score, Damkliang et al. [21] implemented an ensemble approach combining attention-based, traditional, and residual-based U-Net algorithms. The model’s performance was assessed using the Jaccard similarity coefficient, the Dice similarity coefficient, and the mean Intersection over Union (IoU). The ARU-Net model achieved Jaccard coefficients of 0.81 for validation and 0.74 for testing. The mean IoU scores for both benign and malignant classes showed good performance. When evaluating the task of malignant segmentation, the weighted mean ensemble (WME) outperformed the individual models.

For slide 1346, the malignant probabilistic map accurately identified high-probability cancer areas, satisfying pathologists. For slide 1929, re-annotation corrected a misclassified area, improving detection accuracy. The AI-based tool demonstrated the potential to differentiate between benign and malignant prostate tissue in digitized needle biopsy images, aiding in Gleason pattern segmentation [22].

When Gleason variability was compared directly to deep learning platforms, an inter-observer variability of 0.6946 κ was obtained, with a 46% discrepancy in the sizes of the areas annotated by the pathologists. The most thoroughly trained models achieved a κ score of 0.826 ± 0.014 on the test set when trained with data from the same source, reinforcing the expanding supportive capabilities of the learning program, which can provide a second opinion to the pathologists [23]. Shao et al. proposed a risk stratification model based on CNN to correctly reclassify 3.9% of low-risk patients as high-risk and 21.3% of high-risk patients as low-risk patients when compared to Gleason grading systems, highlighting the importance of machine learning (ML) in histopathological image assessment with risk stratification [24].

Fogarty et al. developed a deep learning (DL) model to identify prominent Gleason patterns using a curated data cohort and validated it on an independent dataset. The histology images were divided into 14,509 tiles, with expert annotations for glandular structures and assigned Gleason grades. Transfer learning was utilized for fine-tuning several deep neural network architectures, initially trained on ImageNet, to adapt them to histopathological discrimination. With a 52-patient baseline, the best DL model achieved 91% accuracy, an F1-score of 0.91, and an AUC of 0.96 in distinguishing cancer grade (GS3/4) from benign tissue. Within a 40-patient test, the model achieved 68% accuracy and an AUC of 0.71 for distinguishing between GS3 and GS4. The baseline scores presented by the authors focus on distinguishing primary Gleason patterns in individual glands or small patches of prostate whole slide images (WSI). The study demonstrates that a CNN deep learning (DL) model can accurately discriminate malignant patterns from benign tissue [25].

After an extensive ablation study, a ResNet18 CNN classifier utilizing transfer learning was implemented with the goal of addressing the underlying issues associated with label inconsistencies within Gleason grading accuracy. This was employed through a multi-label deep learning classifier, which implemented an ensemble of three one-vs-all deep models to detect Gleason grades (G3, G4, and G5) in histopathology images. With a 14% increase in accuracy and a 4% rise in the F1-score, the multi-label ensemble classifier emphasized its precision and reliability, further underscoring the potential for machine learning in a clinical setting [26].

### 3.3. Comparative Analysis of Commercial and Public Learning Models for Histopathological Image Analysis

The prostate cancer grade assessment (PANDA) challenge aimed to accelerate medical imaging innovation by allowing the open comparison of various deep-learning models to identify and classify their efficacy to validate the next generation of prostate cancer diagnostics. The competition framework kept developers separate from the independent evaluation process, reducing the risk of information leakage and providing an authentic measure of the algorithms’ diagnostic capabilities. This approach demonstrated that integrating AI with innovative study designs and thorough, predetermined validation across various cohorts can effectively address complex and critical medical challenges [27].

The PANDA challenge assessed various public AI algorithms, including Kiminya, BearlyBears, PND, NS_Pathology, and Vanda. Among them, PND, BarelyBears, and NS_Pathology demonstrated superior performance. Specifically, PND achieved a quadratic weighted kappa (QWK) score of 0.862, BarelyBears scored 0.845, and NS_Pathology had a QWK of 0.760, reflecting high agreement with the pathologists.

Notably, NS_Pathology excelled in distinguishing tumors from benign tissue, with a specificity of 94.50% and a sensitivity of 93.80%. At a clinically relevant threshold combining benign tissue and Grade Group 1, NS_Pathology maintained high performance, with a specificity of 91.50% and a sensitivity of 84.90%. PND and BarelyBears also performed well, with PND achieving a specificity of 75.00% and sensitivity of 98.40%, while BarelyBears achieved a specificity of 82.70% and a sensitivity of 96.70%. At a different threshold, PND showed a specificity of 80.30% and a sensitivity of 95.20%, while BarelyBears displayed a specificity of 76.10% and a sensitivity of 100% [28].

Commercial algorithms, such as Paige Prostate and AIRA Prostate, have shown promising results and underscore the potential of AI technology in clinical settings. Notably, the Paige Prostate AI has received FDA approval for use, highlighting its clinical applicability [29]. Paige achieved a specificity of 87.90% at a sensitivity of 98.80%, while AIRA Matrix demonstrated a higher specificity of 91.80% at the same sensitivity of 98.80%. At the clinically relevant threshold where benign tissue and Grade Group 1 are considered versus all other categories, Paige attained a specificity of 92.30% with a sensitivity of 93.40%, and AIRA Matrix demonstrated a specificity of 90.40% with the same sensitivity of 93.40%. 

Further, when evaluating the clinically relevant threshold of benign tissue, Grade Group 1 and Grade Group 2 versus Grade Groups 3, 4, and 5, the AIRA Matrix reached a specificity of 94.40% with a sensitivity of 92.90%. In contrast, Paige achieved a perfect specificity of 100% but with a lower sensitivity of 71.40%. These results demonstrate the efficacy of commercial AI algorithms in accurately identifying prostate cancer patterns, further supporting their potential role within clinical decision making [28].

Commercial algorithms achieved the highest performance overall, with the best-performing public algorithms demonstrating comparable results as their commercial counterparts. On average, commercial algorithms exhibited a higher agreement with pathologists compared to public algorithms on an identical dataset. When we look at commercial algorithms in comparison, they tend to underestimate cases more frequently than academic ones. The discrepancy may be caused by the difference in optimization goals. The PANDA challenge algorithms were specifically optimized for the kappa coefficient, while commercial algorithms were optimized for clinical decision-making.

### 3.4. Multi-Omic and Radiomic Features for Gleason Pattern Prediction

Modern systems biology, leveraging “omics” technologies, holds great promise for minimizing difficulties presented in the modern era of precision medicine. The application of “omic” techniques to cancer research represents a ground-breaking approach for identifying new biomarkers. This methodology could result in the discovery of unique biomarker molecules and molecular signatures with promising clinical relevance [30].

Researchers utilize such technology to capture diverse molecular characteristics of biological systems with high throughput and resolution, resulting in the creation of extensive and intricate datasets. By combining these various sources of biological data, we can gain a deeper understanding of complex diseases like cancer. However, a significant challenge lies in integrating the heterogeneous data produced into a single machine-learning model for effective classification. A PaCMAP-embedded CNN for multi-omics data integration was proposed by Qattous et al. [31], involving a novel approach that combines copy number alteration (CNA), DNA methylation, and gene expression data to forecast the Gleason score.

Scientists use this technology to efficiently and precisely record a wide range of molecular features of biological systems, generating large and complex datasets. Through the integration of many biological data sources, we may enhance our comprehension of complex diseases such as cancer. Despite this, the complexities of diverse data merging into a unified machine learning model for efficient categorization remains, and Qattous et al. presented a PaCMAP-embedded CNN for multi-omic data integration through the incorporation of copy number alteration (CNA), DNA methylation, and gene expression data to predict the Gleason score.

The model demonstrated a superior performance in comparison to the state-of-the-art i-SOM-GSN model by integrating multi-omics data with a CNN architecture, achieving an accuracy of 98.89% and an AUC of 0.9996. These results highlight the advanced role and reliability of multi-omic data for predicting patient outcomes. A combination of PaCMAP with RBG and a CNN assist in reducing the number of dimensions while enhancing visualization and prediction technology, creating a robust framework for accurate prediction encompassing different types of omics data [31].

Innovative radiomic features allow for the extraction of biological data from standard MRI image sequences. An independent study aimed to develop a new model using the joint intensity matrix (JIM) to predict the Gleason score in prostate cancer patients. Five JIM-derived features, including contrast, homogeneity, difference variance, dissimilarity, and inverse difference, were found to be independent predictors of GS (*p* < 0.05). The combination of JIM and GLCM analyses yielded the highest AUC values: 78.40% for GS ≤ 6, 82.35% for GS = 3 + 4, and 64.76% for GS ≥ 4 + 3, emphasizing the complementary value of JIM features to the gray level co-occurrence matrix [32].

Through multi-omics, Ning et al. [33] utilized the integration of biological domains to provide a well-defined insight into prostate cancer that could predict the Gleason grading of prostate cancer, facilitating precise patient stratification. By combining data from radiomics, genomics, and proteomics, the study analyzed 146 newly diagnosed prostate cancer patients who underwent PET/MR scans prior to radical prostatectomy. Isolation of DNA was carried out through formalin-fixed paraffin-embedded tissue (FPPE) samples from prostatectomy, and whole exome sequencing was conducted.

Through multi-omics, Ning et al. utilized the integration of biological domains to provide a well-defined insight into prostate cancer that could predict the Gleason grading of prostate cancer, facilitating precise patient stratification. By combining data from radiomics, genomics, and proteomics, the study analyzed 146 newly diagnosed prostate cancer patients who underwent PET/MR scans prior to radical prostatectomy.

Regarding proteomics, an immunohistochemical analysis was completed with PCa-specific biomarkers. Among various ML-based approaches, the explainable boosting machine (EBM) algorithm performed best, achieving a sensitivity of 75.00% and a specificity of 88.00%, a PPV of 75.00%, an NPV of 88.00%, an accuracy of 83.00%, and an AUC of 0.81. The robustness of these metrics was confirmed using the 10-fold Monte Carlo cross-validation. In comparison to needle biopsy for predicting Gleason grading, the EBM model improved the sensitivity by 13%, NPV by 7%, accuracy by 2%, and the AUC by 4%. Conversely, the specificity and PPV decreased by 4% and 6%, respectively. These results demonstrate the superior accuracy of multi-omics-based machine learning in Gleason grading compared to the current clinical baseline. Its use can target and refine clinical decision-making, alongside personalized prostate cancer management [33].

Emerging studies have utilized machine learning to analyze radiomic features derived from B-mode ultrasound, shear-wave elastography (SWE), and dynamic contrast-enhanced ultrasound (DCE-US) to localize prostate cancer lesions. Prior to surgery, 50 men with biopsy-confirmed PCa underwent transrectal ultrasound, SWE, and DCE-US. The images were automatically segmented and registered, generating radiomic features from all imaging modalities. A random forest classification algorithm was applied to these images. The study reported an AUC of 0.75 for detecting PCa and an AUC of 0.90 for detection of significant prostate cancer with Gleason scoring > 3 + 4 [34].

The benefit of a supplementary assessment through AI could alleviate the workload of pathologists and permit the evaluation of more patients. When Ström et al. used deep neural networks to evaluate the grading of 1631 biopsies, it achieved an AUC of 0.997 when distinguishing between malignant and benign tissues in an independent dataset. Alongside this, an AUC of 0.986 was resultant in the external validation dataset [35].

To summarize our findings, we collected and displayed some of the intelligent models in Table 1.

### 3.5. Ethical and Practical Considerations in Implementing AI for Prostate Cancer Diagnostics

The practicality of the SRS platform permits easy tumor detection due to its use of rapid Gleason scoring on prostate needle core biopsies. Given that it does not require complex tissue processing, it enables a more streamlined process. Despite this, we must address further hindrances, such as the bulky configuration of the solid-state lasers and expanding the training dataset to proceed with the aim of clinical implementation [11].

A secondary problem in prostate cancer diagnosis arises in observer inconsistency. Reliability is hindered when using single pathologists to annotate both the training and test datasets, or using a single slide scanner to generate digital slides can negatively affect the reliability of the assessment. Furthermore, with no standardization of input image dimension for the deep learning models, ResNet was fed small input images with more detail, resulting in feature generation that poorly depicted the actual tissue detail [13].

Some studies obtained the dataset from an automated grading method rather than from expert pathologists. While this reduces the inter- and intra-pathologist variability in Gleason grading, this approach may not be able to capture the intricacies of expert annotations. The MRI–histopathology system has inherent errors of approximately 2 mm on the prostate border and 3 mm inside the prostate, which could affect the small lesions. In addition to this, limiting the model to certain datasets creates an issue of a wider detection capability with patients who may not be included in the specific demographic of results for which the model is trained [15].

The lack of data also limited Schmidt et al., leaving the algorithm unable to predict prostate cancer death in men who underwent RP. Furthermore, an emphasis on GG creates a secondary problem of the cancer itself due to the lack of focus and attention on outcomes such as metastasis or biological recurrence. The practical significance and applicability of the healthcare findings were constrained and uncertain, given that the study did not evaluate the real-world impact of the AI algorithm. Although efforts were made to manually select tiles to guarantee diversity, there remains a risk of potential selection bias [17].

Generalizability also poses several weaknesses in design. Not only does using one pathologist limit the reliability of the test results, but the study performed by Jung et al. only evaluated the total duration of examining every 30 cases, not the duration of examining each slide. Given that the differences at a granular level were not assessed, it would inevitably affect the precision and applicability of the test results [18].

Damkliang et al. were presented with a recurring issue of overfitting, resulting from the incapability of analyzing new data despite performing well on the training data. This raises another concern: The model learns all the nuances, including the noise in the training data, which compromises its efficacy when applied to new data. Despite the models’ successes in malignant tissue localization and identification, the binary basis training inhibited them from correctly differentiating between GS, GP, and GG [22].

The scarcity of samples issues a challenge when attempting to accurately identify more complex patterns, for example, when higher-grade Gleason pattern displays distinct features, such as the reduced luminal size, and the model faces challenges to learn and distinguish these patterns. This was predominantly seen in the PANDA Radboud public dataset. however, it should be noted that some patches might contain multiple glands [25].

Some studies need further validation before they can be applied to clinical contexts. The exclusion of pertinent tumor features, like convexity, volume, solidity, and eccentricity, could potentially introduce bias due to manual segmentation. The use of publicly available data sources with absent or limited supplementary clinical information, such as PSA and MRI PI-RADS data, could be another reliability threat [32].

Despite the vast utility of radiomics, only a small subset of research is considered relevant. The use of systematic biopsies in Wildeboer et al.’s [34] investigation is prone to errors that could impact the SWE and DCE-US results. Alongside this, the study opted toward the cognitive reading of images and videos rather than utilizing an automated pixel value-based method. Moreover, the study was conducted at a single center and involved only 50 patients with biopsy-proven prostate cancer, but validating the diagnostic efficacy of machine learning across diverse patient populations requires prospective, multicenter research [34].

Despite the vast utility of radiomics, only a small subset of research is considered relevant. The use of systematic biopsies in Wildeboer et al.’s [34] investigation is prone to errors that could impact the SWE and DCE-US results. Alongside this, the study opted toward the cognitive reading of images and videos rather than utilizing an automated pixel value-based method.

## 4. Discussion

The central question of whether AI should be relied upon when making clinical decisions in patient care, particularly in prostate cancer diagnosis, remains complex and multifaceted. On one hand, AI models, such as convolutional neural networks (CNNs), residual networks (ResNets), and U-Net architectures, have demonstrated significant promise by achieving high accuracy rates, often comparable to or exceeding those of human pathologists. For example, the DeepDx algorithm achieved a Cohen’s kappa score of 0.91, indicating a strong agreement with pathologists, and demonstrated a remarkable accuracy of 96% in distinguishing between malignant and benign cases, with a sensitivity of 99.7% and specificity of 88% [17]. Similarly, ensemble and residual-based approaches, such as the 101-layer ResNet models, have achieved accuracies of 88% to 89% in differentiating cancerous from non-cancerous tissues [12,13]. Moreover, hybrid models, like CorrSigNIA, which integrates multi-modal imaging data from MRI and whole-mount histopathology, have been shown to achieve over 80% accuracy in cancer identification, highlighting the potential of AI to standardize Gleason grading, reduce inter-observer variability, and improve diagnostic efficiency, thereby potentially enhancing patient outcomes [15].

However, despite these advancements, several significant challenges still hinder the full clinical adoption of AI in prostate cancer diagnostics. A major concern is the variability and quality of the data used to train these models. For instance, many studies rely on relatively small and potentially biased datasets, which limits the generalizability of the AI models across diverse populations. The PaCMAP-embedded CNN, while achieving high accuracy and AUC scores (98.89% and 0.9996, respectively) through the integration of multi-omics data, like copy number alterations, DNA methylation, and gene expression data, is still constrained by the quality and diversity of its input data [31]. Similarly, models trained on datasets with limited demographic representation, such as those seen in the Prostate Cancer Grade Assessment (PANDA) challenge, may not perform as well in different clinical settings. Although the PANDA challenge fostered innovation by comparing deep-learning models, the models’ performance showed significant variability, with commercial algorithms like Paige Prostate and AIRA Prostate achieving high specificity (87.9%) and sensitivity (98.8%) but also demonstrating tendencies to underestimate certain cases more frequently than academic models. These findings underscore the limitations of relying on a limited set of data sources and emphasize the need for broader, more diverse datasets to improve AI reliability in clinical practice [27,28].

Further complicating the question of AI reliance in clinical decision-making is the issue of diagnostic inconsistencies and observer variability. For example, studies using stimulated Raman scattering (SRS) microscopy for automated Gleason pattern classification have achieved high accuracy rates (85.7% for classification and 84.4% on validation cases) yet still demonstrated only 71% consistency with pathologists’ scores, suggesting that AI systems may not yet be fully reliable for sole diagnostic use [11]. Similarly, while deep learning models, like the Automated Tumor Assessment of Prostate Cancer Histology (ATARI) and hybrid architectures such as those employing EfficientNet, have shown strong performance metrics, their diagnostic consistency may still vary significantly depending on the quality and source of training data [12,13,14]. Issues such as feeding models small input images with more detail, which can result in feature generation that poorly depicts actual tissue characteristics, further complicate the use of AI in clinical settings. Moreover, problems such as overfitting, where models like those used by Damkliang et al. perform well on training data but struggle with new data, raise concerns about the generalizability of AI models to real-world clinical environments. This is particularly important in the context of higher-grade Gleason patterns, where AI models have difficulty distinguishing between complex patterns due to limited sample sizes and inherent biases within the training datasets, as seen in studies utilizing the PANDA Radboud public dataset [22,28].

The integration of AI into clinical workflows is also challenged by technical limitations and a lack of standardization. For instance, the configuration of hardware used in AI systems, such as the bulky solid-state lasers in SRS platforms, can be impractical for routine clinical use [11]. Moreover, the lack of standardization in input image dimensions for deep learning models, such as ResNet being trained on small, detailed images, can lead to suboptimal feature extraction and reduced accuracy [13]. Additionally, while AI models can help standardize certain aspects of prostate cancer grading, they may lack the nuanced interpretative skills of experienced pathologists. For example, models that utilize automated grading methods rather than expert annotations may fail to capture the intricate details of tissue morphology that expert pathologists can recognize, potentially affecting the accuracy and reliability of the diagnosis. Moreover, limitations in the accuracy of MRI–histopathology correlations, such as inherent errors in prostate border delineation (approximately 2 mm) and inside the prostate (approximately 3 mm), can introduce further inaccuracies, particularly when detecting small lesions [15]. In addition, several studies have highlighted the need for further validation before AI models can be applied broadly in clinical contexts. The exclusion of critical tumor features, like convexity, volume, solidity, and eccentricity, from some AI models could introduce bias due to manual segmentation, while the use of public datasets with limited or missing supplementary clinical information, such as PSA and MRI PI-RADS data, threatens the reliability of the AI systems [32]. The lack of large, multicenter validation studies also limits the ability to assess AI efficacy across diverse patient populations. As illustrated by the study conducted by Wildeboer et al., which used systematic biopsies prone to errors, the small sample size (50 patients) and single-center design underscore the need for prospective, multicenter research to validate AI performance across diverse clinical settings [34].

Thus, while AI has demonstrated potential to transform clinical decision-making in prostate cancer diagnosis by improving diagnostic accuracy and reducing observer variability, it should currently be regarded as a complementary tool rather than a replacement for human expertise. The limitations associated with data quality, model generalizability, technical constraints, and the need for robust validation necessitate a cautious approach to AI integration in clinical practice. Moving forward, the focus should be on developing more robust, diverse datasets, enhancing model interpretability, and standardizing protocols to ensure that AI systems provide both safe and effective in-patient care. Only then can AI’s full potential be realized, providing a reliable support tool that augments, rather than replaces, the critical judgment of experienced clinicians.

## 5. Conclusions

The role of AI algorithms in identifying Gleason patterns in prostate cancer is growing. Recent findings have revealed that AI algorithms can make valuable contributions to traditional diagnostic methods. These findings underscore the potential for integration of AI into clinical practice, improving the workflow of pathologists and enhancing patient outcomes. AI’s potential as an auxiliary tool for Gleason grading can help combat inter-observer variability. Despite the scientific leap in the technological applications of AI targeted for patient care, there remains key issues that must be addressed to ensure the smooth transition from clinical trials to clinical practice. Sample sizes and overfitting remain some of the most vulnerable areas of AI, drawing attention to the infancy and prematurity of the program. Given time, the datasets will continue to facilitate more patient information, allowing the AI to be trained on more diverse information, facilitating its purpose in the era of precision medicine.

## Figures and Tables

**Table 1:** Inteligent models used for Identification and Comparison of Gleason Patterns in Prostate Cancer Histopathology.

Study	Objective	Study Design	Algorithm/Model	Results
Ao et al. [11]	Gleason assorting of prostate needle biopsy and automatic diagnosis	Images from 61 patients Validation with 22 independent cases Gleason scores of core needle biopsies from 21 cases	CNN Deep learning-based SRS	CNN: 85.7% Validation of independent case accuracy: 84.4% Deep learning-based SRS: 71% diagnostic consistency with 3 pathologists
Duenweg et al. [13]	Assess ML models for differentiating cancerous regions and Gleason patterns in prostate histology	Digitized histology from 47 PCa patients, training set of 31 patients, and testing set of 16 patients	ResNet model Bagged ensemble model	ResNet accuracy: 88% Bagged ensemble model: 89% Comparison: ResNet > Bagged ensemble model
Bhattacharya et al. [15]	Integrate MRI and histopathology images to differentiate aggressive and indolent prostate cancers	Evaluation on RP and biopsy cohorts; 3 independent test sets	CorrSigNIA: CNN with correlated MRI and histopathology features	Accuracy: 80% ROC-AUC of general cancer detection: 0.81 ROC-AUC of clinically significant cancer: 0.82–0.86
Schmidt et al. [17]	Validate DeepDx prostate AI for Gleason grading on whole-mount prostate histopathology	500 tiles from 150 whole-mount RP specimens	DeepDx prostate AI, trained on biopsy images	Cancer detection: Cohen’s kappa of 0.91 Gleason grading: Cohen’s kappa of 0.89 Cancer detection: SNS of 0.997 and SPC: 0.88 GG classification: SNS of 0.98 and SPC of 0.85
Jung et al. [18]	Validate DeepDx performance in prostate cancer diagnosis and grading	593 whole-slide images of prostate biopsy; 130 normal and 463 adenocarcinomas	DeepDx prostate AI, artificial neural network-based diagnostics system	GGs kappa/quadratic-weighted kappa: 0.713/0.922 GSs kappa/quadratic-weighted kappa: 0.654/0.904
Zhang et al. [21]	Develop AI system to diagnose prostate cancer from histopathological specimen	896 whole-mount sections from 160 patients. 826 sections from 148 patients in training set Testing set section from 12 patients	Modified U-Net for the image segmentation	ROC-AUC: 96.8% Pixel accuracy: 96.93% (binary), 93.88% (clinically oriented), 93.88% (ISUP Gleason grade) AI and pathologist agreement on diagnosis of 21/22 sections
Fogarty et al. [25]	Develop a DL model to analyze Gleason patterns in prostate cancer	Histology images sectioned into 14,509 tiles	Deep neural network trained on ImageNet improved with histology	GS3/4 vs benign: 91% accuracy, 0.91 F1-score, 0.96 AUC from 52 patients GS3 vs GS4: 68% accuracy, 0.71 AUC from 40 patients
Faryna et al. [28]	Comparison of the performance of public and commercial AI Gleason grading algorithm	Whole-slide prostate biopsy images including 5 public algorithms from PANDA and 2 from commercial algorithms 10 pathologists	Public algorithms from PANDA challenge and 2 commercial Gleason grading algorithms	Pathologists’ quadratic kappa: 0.777–0.916 AI algorithms’ quadratic kappa: 0.617–0.900 Comparison: commercial algorithms > public algorithms
Qattous et al. [31]	Multi-omics data integration for Gleason scoring to enhance prognostics in cancer diagnosis	Integration of CNA, DNA methylation and gene expression data using PaCMAP RGB for visualization CNN for prediction	CNN for prediction and PaCMAP for dimensionality reduction	Accuracy: 98.89% AUC: 0.9996 Comparison: outperforms the i-SOM-GSN model
Ning et al. [33]	Prediction of Gleason grading utilizing multi-omics data	Retrospective study of 146 patients with PCa with integration of radiomics, genomics and proteomics data	U-net-based segmentation for radiomics Whole exome sequencing for genomics Immunohistochemistry for proteomics EBM classification for the integration	EBM model: SNS of 0.75, SPC of 0.88, PPV of 0.75, NPV of 0.88, ACC of 0.83, ACC of 0.83 and AUC of 0.81 Improvement of the needle biopsy predictions of SNS by 13%, NPV by 7%, ACC by 2% and AUC by 4%
Ström et al. [35]	Develop an AI system for detection of prostate cancer, localization, and Gleason grading	Digitized 6682 biopsy slides from 976 participants and 271 slides from 93 men 23 pathologists	Deep neural networks for detecting the presence, extent and Gleason grading for malignant tissues	Cancer detection on test dataset: AI achieved AUC of 0.997 and 0.986 on external dataset Gleason grading with expert pathologist: Kappa of 0.62 AI’s prediction of cancer length correlated at 0.96 with pathologist measurements

## Data Availability

Data available on request due to privacy restrictions.
